# Händehygiene im OP – (k)ein Thema?

**DOI:** 10.1007/s00113-022-01181-0

**Published:** 2022-05-02

**Authors:** Justus Menzel, Annika Kühn, Diana Beck, Bettina Schock, Iris F. Chaberny

**Affiliations:** Institut für Hygiene, Krankenhaushygiene und Umweltmedizin, Universitätsklinikum Leipzig AöR, Liebigstr. 22, 04103 Leipzig, Deutschland

**Keywords:** Händehygiene-Compliance, Fortbildungen, Maßgeschneidert, Intervention, Direktes Feedback, Hand hygiene, Tailor-made training, Operating room (OR), Surgeon, Anesthesiologist

## Abstract

**Hintergrund und Zielstellung:**

Eine hohe Händehygiene-Compliance (HHC) ist eine effektive Maßnahme zur Prävention von nosokomialen Infektionen. Die WHO empfiehlt direkte Beobachtungen als Goldstandard, um die HHC zu stratifizieren. Hohe Compliance-Raten bei der chirurgischen Händedesinfektion legen eine hohe Gesamt-Compliance im OP-Bereich nahe. Zahlen zur hygienischen Händedesinfektion im OP sind allerdings rar. Ziel war es, die HHC systematisch zu beobachten und durch maßgeschneiderte Interventionen zu steigern.

**Methode:**

Um die HHC in den Jahren 2017 und 2018 zu erfassen, wurde ein Beobachtungsbogen genutzt. Chirurgen und Op.-Pflege sowie Anästhesisten und Anästhesiepflege wurden erfasst. Für die Erprobung einer maßgeschneiderten Intervention wurden 2 OP-Bereiche ausgewählt. Zur Überprüfung der Effektivität erfolgten nur in einem OP-Bereich eine maßgeschneiderte Fortbildung und sog. Compliance-Begleitungen mit direktem Feedback durch die Hygienefachkräfte. In dem anderen OP-Bereich erfolgte keine Intervention.

**Ergebnis:**

Über 1500 Indikationen zur Händehygiene wurden in den Jahren 2017 und 2018 im operativen Bereich erfasst. Die Gesamt-Compliance der Interventionsgruppe konnte im Beobachtungszeitraum von 40 auf 75 % gesteigert werden (*p* < 0,001). Die Gesamt-Compliance der Kontrollgruppe veränderte sich nicht signifikant (48 auf 55 %; *p* = 0,069).

**Diskussion:**

Durch die hohe Compliance-Rate bei der chirurgischen Händedesinfektion entstand die Annahme, die Compliance bei der hygienischen Händedesinfektion sei ebenfalls auf einem hohen Niveau. Im Rahmen der Feedbackgespräche zeigte sich, dass die Mitarbeiter nicht wussten, dass die Indikationen der „5 Momente der Händehygiene“ auch im OP anzuwenden seien, und zeigten somit ihr Unwissen über die assoziierten Indikationen.

## Abstract (en)

**Background and objective of the study:**

In the knowledge that hand hygiene is a key measure in preventing healthcare-associated infections, the WHO recommends direct observation as the gold standard in order to evaluate compliance. High compliance rates when performing surgical hand disinfection imply a high rate of compliance throughout the operating room (OR). Concrete numbers reflecting hand hygiene within the OR are rare, however, which is why our goal was to systematically observe hand hygiene and create tailor-made training solutions in order to improve it.

**Methods:**

A hand hygiene data collection form was used to document compliance observations in the OR in 2017 and 2018. Compliance was monitored in two separate surgical departments. Surgeons and perioperative nurses, as well as anesthesiologists and nurse anesthetists, were observed. In order to test a tailor-made training solution, two separate surgical departments were chosen. To test the effectiveness of the training solution, only one of the two surgical departments received a tailor-made training along with direct feedback from a trained infection control nurse. In the second surgical department, no training intervention took place.

**Results:**

More than 1500 indications for hand hygiene were observed in the OR between 2017 and 2018. Overall compliance in the intervention group increased from 40% to 75% during the observation period (*p* < 0.001). Overall compliance in the control group did not increase significantly (48% to 55%; *p* = 0.069).

**Discussion:**

Given that the compliance rate for surgical hand disinfection is so high, the assumption was that the compliance for routine hand hygiene within the OR would be similar. Within the framework of the feedback talks, it became apparent that the employees were unaware that the “5 moments for hand hygiene” also apply within the OR. The employees were also unaware of what exactly the five indications were.

## Hinführung zum Thema

Der OP gilt als einer der „reinsten“ Orte eines Krankenhauses, ausgezeichnet durch bauliche Abgrenzung und besondere Anforderungen. Die Operateure und die „sterile“ Assistenz führen präoperativ eine chirurgische Händedesinfektion durch, bevor sie die sterile Schutzkleidung anlegen und die Operation beginnt. Steriles Arbeiten hat intraoperativ höchste Priorität. Doch neben der eigentlichen Op. erfolgen perioperativ verschiedene Handlungen am Patienten, deren Relevanz für eine Erregerweiterverbreitung möglicherweise unterschätzt wird. Die beteiligten Berufsgruppen und Fachdisziplinen werden hinsichtlich hygienischer Aspekte bei solchen perioperativen Tätigkeiten im Folgenden genauer unter die Lupe genommen.

## Einleitung

Der Operationsbereich aller chirurgischen Fächer ist aus hygienischer und infektionspräventiver Sicht ein sehr sensibler Ort bei der Entstehung und zugleich auch bei der Prävention von nosokomialen Infektionen (NI) [1]. Die hygienische Händedesinfektion gilt nach wie vor als die wirksamste, einfachste und kostengünstigste Maßnahme zur Reduzierung von NI [2].

Ziele aller Präventionsmaßnahmen bei invasiven Eingriffen sind der Schutz von Patientinnen und Patienten sowie der Schutz von Mitarbeiterinnen und Mitarbeitern vor nosokomialen bzw. berufsbedingten Infektionen. Zu einem wirksamen Infektionsschutz tragen persönliche Verhaltensweisen sowie patientenbezogene spezifische Schutzmaßnahmen bei [3]. Hierzu zählen neben der hygienischen Händedesinfektion (HHD), welche den indikationsgerechten Einsatz von Einmalhandschuhen beinhaltet, und der chirurgischen Händedesinfektion das korrekte Tragen eines Mund-Nasen-Schutzes (MNS) und einer Op.-Haube [4].

Während über den Einfluss von technischen Ausstattungen von OP, z. B. raumlufttechnischen Anlagen (RLTA), in der Infektionsentstehung bereits Untersuchungen existieren [5], ist die Datenlage zur Relevanz von perioperativen Prozessen im OP als primäre Infektionsquellen bisher gering. Ein großer Fokus wird auf sterile Kautelen im OP gelegt, wobei die Entstehung von NI durch Erregerübertragungen bei direktem Patientenkontakt perioperativ durch das medizinische Personal und die Praktiken der Händehygiene weitgehend unbeachtet blieben, obwohl die Hände als Quelle von Erregertransmissionen identifiziert sind [6]. Auch Daten zur HHC des medizinischen Personals im OP und deren Rolle bei der Prävention von NI sind in der Literatur bisher rar, wenngleich Compliance-Verstöße bei den im OP tätigen Berufsgruppen bereits identifiziert werden konnten [7] und durch eine Steigerung der HHC die Prävalenz von NI gesenkt werden konnte [8–10].

Ziel der vorliegenden Studie war es, die HHC im OP-Bereich zu erfassen und durch maßgeschneiderte Interventionen eine Verbesserung des Hygieneverhaltens zu erreichen.

## Methode

**Figure 1:**
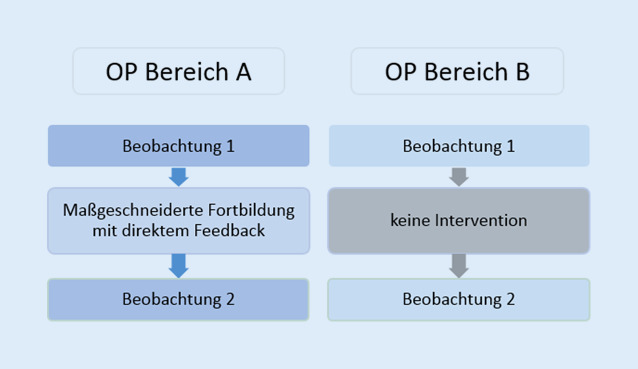

In den Jahren 2017 und 2018 erfolgte die prospektive Erfassung der HHC in 2 baulich getrennten Operationsbereichen unterschiedlicher operativer Fachbereiche des Universitätsklinikums Leipzig AöR analog zu der Aktion Saubere Hände (ASH), welche die Compliance im stationären Bereich anhand der 5 Momente der Händehygiene (5MdHH) systematisch erfasst [11]. Um den Effekt von maßgeschneiderten Interventionen zur Verbesserung der Compliance zu zeigen, wurden im OP-Bereich A zum einen eine maßgeschneiderte Fortbildung sowie Compliance-Begleitungen mit direktem Feedback über einen Zeitraum von 4 Wochen durchgeführt. Das Personal der Kontrollgruppe erhielt lediglich die jährliche Pflichtschulung. Wie der Abb. 1 entnommen werden kann, erfolgte eine erneute Beobachtungsphase jeweils ca. 12 Wochen nach der Interventionsphase über einen Zeitraum von 4 bis 6 Wochen. Es wurden ca. 500 Einzelbeobachtungen bei den im OP tätigen Berufsgruppen (Chirurgen, Op.-Pflege, Anästhesisten und Anästhesiepflege) durchgeführt.

### Beobachtungsbogen

Als Erhebungsinstrument diente ein Beobachtungsbogen. Dieser orientiert sich an dem Beobachtungsbogen für die HHC der ASH und erfasst 4 Kategorien: Berufsgruppe (stratifiziert nach Chirurgen, Op.-Pflege, Anästhesisten und Anästhesiepflege), Indikation (gemäß 5MdHH), Zuordnung aseptischer Tätigkeiten und Händedesinfektion.

### Intervention

**Figure 2:**
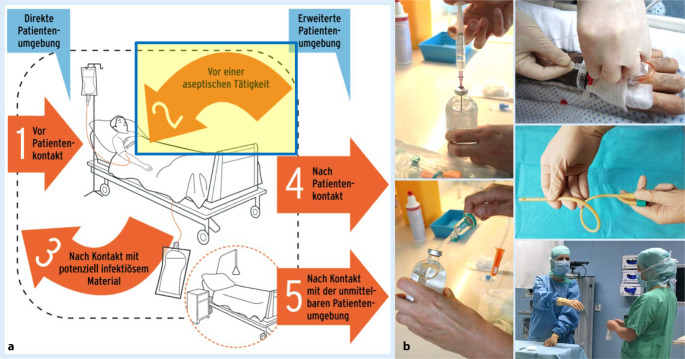

Nach Abschluss der ersten Beobachtungsphase wurde eine maßgeschneiderte Fortbildung so konzipiert, dass die Inhalte der Händehygiene im operativen Bereich genauer und zielgruppenorientiert (für Chirurgen, Op.-Pflege, Anästhesisten und Anästhesiepflege) vermittelt wurden. Unter Verwendung der durch die Compliance-Beobachtung als häufigste Tätigkeiten für das Personal erfassten Anwendungsfälle wurde ein praxisnaher Bezug erstellt (Abb. 2).

Die zur Schulung benutzten Grafiken haben zum Ziel, einen Überblick über die Indikationen der Händedesinfektion im OP zu geben. Zudem sind die aseptischen Tätigkeiten aufgeführt, die durch die Compliance-Beobachtung als häufigste Tätigkeiten für Op.-Personal erfasst worden sind.

### Compliance-Begleitung mit direktem Feedback

Im Anschluss an die maßgeschneiderte Schulung erfolgten Compliance-Begleitungen durch die Hygienefachkräfte (HFK), in denen direktes Feedback zu den 5MdHH in den beobachteten Situationen übermittelt wurde. In Situationen von Hygieneverstößen wurden diese direkt angesprochen, und es wurde versucht, vor Ort gemeinsam Lösungsansätze zu entwickeln, die die gegebenen Arbeitsabläufe, Ressourcen und baulichen Gegebenheiten berücksichtigen.

### Statistische Methoden

Die Datenauswertung erfolgte mithilfe des Statistikprogramms IBM SPSS® Statistics Version 23 (IBM Corporation, Armonk, NY, USA) überwiegend deskriptiv. Bei der Angabe von Prozentwerten wurde auf gültige Prozente zurückgegriffen, bei denen fehlende Werte in die Berechnungen nicht miteinbezogen wurden. Zur Prüfung signifikanter Unterschiede wurde der Chi-Quadrat-Test herangezogen. Das Signifikanzniveau wurde grundsätzlich auf das gängige Niveau von 5 % festgelegt.

## Ergebnisse

### Gelegenheiten für die hygienische Händedesinfektion im OP

Die Gesamtübersicht in Tab. 1 zeigt die *Anzahl an* Gelegenheiten für die hygienische Händedesinfektion in den 2 OP-Bereichen. Bei der Erfassung der Indikationen für die hygienische Händedesinfektion konnten im Zeitraum von 2017 bis 2018 insgesamt 1631 Gelegenheiten dokumentiert werden.

**Table 1:** 

OP-Bereich	Händehygiene-Beobachtungen 2017	Händehygiene-Beobachtungen 2018	Gesamt
OP-Bereich A	*n* = 362	*n* = 512	*n* = 874
OP-Bereich B	*n* = 382	*n* = 375	*n* = 757

Die erfassten Situationen konnten dabei den Berufsgruppen Op.-Ärzte (OP-Bereich A, *n* = 149; OP-Bereich B, *n* = 117), Op.-Pflege (OP-Bereich A, *n* = 237; OP-Bereich B, *n* = 264), Anästhesie-Ärzte (OP-Bereich A, *n* = 163; OP-Bereich B, *n* = 219), Anästhesie-Pflege (OP-Bereich A, *n* = 325; OP-Bereich B, *n* = 157) zugeordnet werden (Tab. 2).

**Table 2:** 

Berufsgruppe		Händehygiene-Beobachtungen 2017	Händehygiene-Beobachtungen 2018
OP-Bereich A	Op.-Ärzte	*n* = 86	*n* = 63
	Op.-Pflege	*n* = 75	*n* = 162
	Anästhesie-Ärzte	*n* = 74	*n* = 89
	Anästhesie-Pflege	*n* = 127	*n* = 198
OP-Bereich B	Op.-Ärzte	*n* = 61	*n* = 56
	Op.-Pflege	*n* = 104	*n* = 160
	Anästhesie-Ärzte	*n* = 169	*n* = 50
	Anästhesie-Pflege	*n* = 48	*n* = 109

### Händehygiene-Compliance vor und nach Intervention

**Figure 3:**
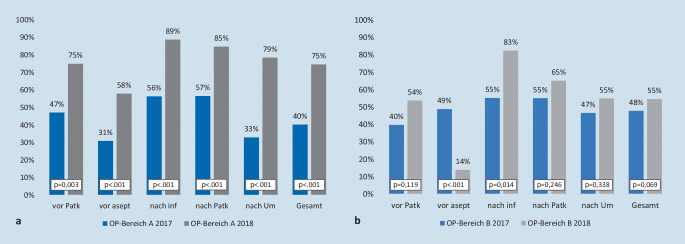

Die Gesamt-HHC im OP-Bereich A lag vor der Intervention bei 40 % und steigerte sich im Jahr 2018 nach erfolgter Intervention signifikant auf 75 % (*p* < 0,001). Die Gesamt-HHC im OP-Bereich B hingegen veränderte sich nicht signifikant (48 % im Jahr 2017 auf 55 % im Jahr 2018; *p* = 0,069). In der Interventionsgruppe konnte die Compliance bei allen 5 Indikationen der Händehygiene gesteigert werden. Am deutlichsten zeigte sich die Verbesserung bei beobachteten Gelegenheiten „nach Kontakt mit der Patientenumgebung“ von 33 auf 79 % (*p* < 0,001). Die Kontrollgruppe ohne Intervention verschlechterte sich bei der Indikation „vor aseptischen Tätigkeiten“ von 49 auf 14 % im Folgejahr (*p* < 0,001; Abb. 3).

### Händehygiene-Compliance im OP, stratifiziert nach Berufsgruppe

**Figure 4:**
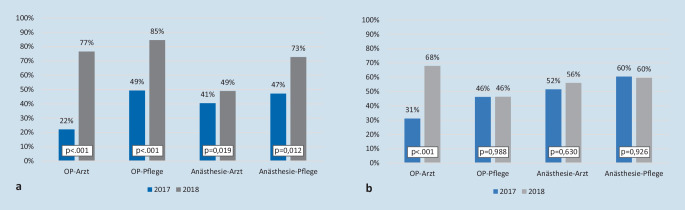

Die Compliance-Raten, stratifiziert nach Berufsgruppen, ist in Abb. 4 dargestellt. Bei der Op.-Pflege steigerte sich die Gesamt-Compliance nach Intervention von 49 auf 85 % (*p* < 0,001). Die Op.-Ärzte in der Interventionsgruppe steigerten ihre Compliance um 55 % (22 % im Jahr 2017 auf 77 % im Jahr 2018; *p* < 0,001). Im gleichen Zeitraum stieg auch die Compliance der Op.-Ärzte in der Kontrollgruppe signifikant von 31 auf 68 % (*p* < 0,001).

## Diskussion

Aufgrund der zahlreichen Empfehlungen und Leitlinien ist es eine Herausforderung, die Inhalte der Hygienefortbildungen interessant und praxisnah zu kommunizieren. Gleichzeitig spielen die Händehygiene und v. a. die Compliance der hygienischen Händedesinfektion die wichtigste Rolle bei der Prävention von NI [9] und werden im Rahmen von Fortbildungen deshalb häufig in den Fokus gerückt. Geht es um die Verbesserung der Händehygiene-Compliance, so wurde der Blick bisher v. a. auf den stationären und weniger auf den operativen Bereich gelegt.

Die vorliegende Studie hat sich das Ziel gesetzt, die HHC im OP-Bereich zu erfassen und eine evtl. Falschannahme der Übertragbarkeit der guten Compliance-Ergebnisse bei der chirurgischen Händedesinfektion aufzuzeigen und durch maßgeschneiderte Interventionen eine Verbesserung der HHC zu erwirken.

Durch die Beobachtungen konnte im Rahmen der ersten Beobachtungsphase erfasst werden, dass nicht wenige Operationen von Anfang bis Ende ohne leitliniengerechtes Händehygieneverhalten verlaufen. Es werden verschiedene perioperative Handlungen mit direktem Patientenkontakt vom Op.-Personal ohne eine einzige Händedesinfektion durchgeführt. So wird beispielsweise der Patient für Lagerungsmaßnahmen berührt, nahe Oberflächen oder das Beatmungsgerät werden angefasst oder verstellt, ohne dass die indizierte Händedesinfektion erfolgt. Nach der Erfassung aller Daten in der ersten Beobachtungsphase wurde deutlich, dass die angenommene HHC sich nicht bestätigen ließ, sondern einen massiven Handlungsbedarf im operativen Bereich erfordert. Durch die Gespräche während der maßgeschneiderten Fortbildung sowie während der Compliance-Begleitung zeigte sich, dass die Mitarbeiter hochmotiviert sind, hygienisch korrekt zu arbeiten, ihnen jedoch die 5 Momente der Händehygiene für den Op.-Kontext keineswegs geläufig waren. Bei der Fortbildung des Op.-Personals zu hygienischen Inhalten konnte so aus den vielversprechenden Erkenntnissen zu maßgeschneiderten Schulungen bei Ärztefortbildungen profitiert werden [12].

Die vorliegenden Ergebnisse zeigen, dass die in der Interventionsgruppe durchgeführten Maßnahmen berufsgruppenübergreifend zu einer signifikanten Steigerung der HHC im OP-Bereich A im Folgejahr geführt haben.

In der Kontrollgruppe zeigte sich eine signifikante Verbesserung der HHC nur in der Gruppe der Op.-Ärzte. Die Autoren gehen davon aus, dass aufgrund der sich überschneidenden Arbeitsbereiche einiger Op.-Ärzte zwischen OP-Bereich A und B ein Informationstransfer stattgefunden hat, durch den sich die Verbesserung der HHC auch in der Kontrollgruppe erklären lässt.

Studien belegen die Relevanz eines Feedbacks zu den erfassten Daten [13]. Im Rahmen von Fortbildungen ist es wichtig, die Mitarbeiter bezüglich der Entwicklung eines Bewusstseins für Infektionsprozesse zu unterstützen, denn nur, wenn dieses Bewusstsein vorhanden ist, ergreifen sie selbstständig spezifische Schritte, um eine Infektion zu verhindern. Zum einen sind dazu Händehygienefortbildungen erforderlich, da das vermittelte Wissen das individuelle Verhalten der Mitarbeiter maßgeblich beeinflusst und existierende Wissenslücken geschlossen werden können [14]. Zum anderen kann eine Verhaltensänderung aber auch durch ein Feedback zur HHC und zu den Infektionsraten unterstützt werden. Die Implementierung von Händehygienefortbildungen in Verbindung mit Feedbackgesprächen hat dabei nachweislich zu einem signifikanten Anstieg der Händehygiene-Compliance und zu einer Reduktion von NI geführt [8]. Die Implementation derartiger Ansätze kann ebenfalls dem Wunsch nach einem erhöhten praktischen Nutzen – über die Händehygiene hinaus – entsprechen und das gewünschte Verhalten im OP fördern.

Die in dieser Studie dargestellte deutliche Verbesserung der HHC innerhalb der Interventionsgruppe lässt auf die hohe Motivation des Personals zur korrekten Durchführung der HHC schließen und macht deutlich, wie wichtig es auch in Zukunft ist, die Indikationen der HHC im OP-Bereich sowie in weiteren klinischen Arbeitsbereichen zu vermitteln, um so eine höhere Patientensicherheit zu gewährleisten.

## Limitationen

Es handelt sich um eine Studie mit der Zielgruppe Personal. Die Studie sammelt keine Daten zu NI innerhalb der beobachteten Patientengruppen. Daher sind Rückschlüsse der verbesserten HHC auf einen Rückgang von NI nicht möglich. Der Zusammenhang von einer gesteigerten HHC auf die Reduktion von NI konnte in anderen Arbeiten bereits gezeigt werden [8, 15].

Im zweiten Beobachtungszeitraum erfolgten in der Interventionsgruppe 137 Beobachtungen mehr als in der Kontrollgruppe. Dies ist auf ein gestiegenes Interesse an den Hygienebegleitungen bei der Interventionsgruppe zurückzuführen, welches mit dem Wunsch nach weiteren Begleitungen durch die HFK verbunden war. Aufgrund begrenzter Ressourcen der HFK erfolgte dadurch eine Fokussierung auf die Interventionsgruppe.

Aufgrund des kurzen Beobachtungszeitraumes lässt sich keine Aussage über die Nachhaltigkeit der erfolgten Intervention treffen. Ziel weiterer Studien muss es sein, auch langfristige Effekt auf das Hygieneverhalten des Op.-Personals zu untersuchen, wenngleich Hinweise auf einen langfristigen Effekt vorliegen [16–18].

Da es sich bei der vorliegenden Studie um eine Beobachtungsstudie handelt, ist davon auszugehen, dass ein Beobachtungsbias im Sinne des Hawthorne-Effektes gruppenübergreifend auftritt und die Compliance-Raten in dieser Arbeit erhöht dargestellt sind [19].

Auf eine Offenlegung der beobachteten Fachbereiche innerhalb des Universitätsklinikums Leipzig wurde bewusst verzichtet, um im Sinne einer positiven Fehlerkultur die weitere Zusammenarbeit der operativen Fachbereiche mit dem Institut für Hygiene, Krankenhaushygiene und Umweltmedizin nicht zu gefährden.

## Zusammenfassend

Händehygiene im OP ist aus infektionspräventiver Sicht nur ein kleiner Bestandteil. Es sollten weitere Aspekte des Verhaltens von Op.-Personal sowie patientenbezogene spezifische Schutzmaßnahmen im OP untersucht werden, um nicht von ähnlichen Falschannahmen auszugehen und sich in hygienischer und damit infektionspräventiver Sicherheit zu wiegen. Erst durch eine umfassende und standardisierte Evaluation aller Präventionsmaßnahmen auch über den operativen Bereich hinaus kann man zu der Annahme kommen, dass einer der sensibelsten Orte im Krankenhaus tatsächlich auch infektionspräventiv gemanagt wird. Der Druck, mittels guter OP-Organisation qualitativ hochwertige und kosteneffiziente Operationen im Einklang mit der Gesamtkrankenhausleistung zu erbringen, steigt massiv und wird letztlich auch ein wesentlicher Teil der Zukunftssicherung sein. Warum dabei nicht gleich die hygienischen Aspekte grundlegend im Blick haben?

## Fazit für die Praxis

- *Maßgeschneidert*: Maßgeschneiderte Fortbildungen im operativen Bereich führen zu einer deutlichen Steigerung der HHC. Der Mehraufwand gegenüber normalen Fortbildungen durch die zielgruppengerechte Anpassung der Inhalte an die Anforderungen in den Risikobereichen lohnt sich.
- *Direktes Feedback geben*: Direktes Feedback zur HHC bietet die Möglichkeit, Hygienefortbildungen mit maximalem Praxisbezug zu gewährleisten. Lösungsansätze können so gemeinsam vor Ort entwickelt werden.
- *Äpfel sind nicht Birnen*: Die hohe Compliance bei der chirurgischen Händedesinfektion ist kein Parameter für eine hohe HHC. Es müssen zukünftig also auch weitere Hygieneaspekte und Präventionsmaßnahmen (z. B. Mund-Nasen-Schutz, Op.-Haube, Handschuhwechsel etc.) im OP beobachtet werden.
